# GPR55 Antagonist CID16020046 Protects against Atherosclerosis Development in Mice by Inhibiting Monocyte Adhesion and Mac-1 Expression

**DOI:** 10.3390/ijms222313084

**Published:** 2021-12-03

**Authors:** Seung-Jin Lee, Dong-Soon Im

**Affiliations:** 1 Department of Pharmacy, College of Pharmacy, Pusan National University, Busan 46241, Korea; leesj@pusan.ac.kr; 2 East West Pharmaceutical Research Center, Department of Biomedical and Pharmaceutical Sciences, Graduate School, Kyung Hee University, Seoul 02447, Korea

**Keywords:** GPR55, CID16020046, atherosclerosis, monocyte adhesion, Mac-1

## Abstract

GPR55 recognizes several lipid molecules such as lysophosphatidylinositol. GPR55 expression was reported in human monocytes. However, its role in monocyte adhesion and atherosclerosis development has not been studied. The role of GPR55 in monocyte adhesion and atherosclerosis development was investigated in human THP-1 monocytes and ApoE^−/−^ mice using O-1602 (a potent agonist of GPR55) and CID16020046 (a specific GPR55 antagonist). O-1602 treatment significantly increased monocyte adhesion to human umbilical vein endothelial cells, and the O-1602-induced adhesion was inhibited by treatment with CID16020046. O-1602 induced the expression of Mac-1 adhesion molecules, whereas CID16020046 inhibited this induction. Analysis of the promoter region of *Mac-1* elucidated the binding sites of AP-1 and NF-κB between nucleotides −750 and −503 as GPR55 responsive elements. O-1602 induction of Mac-1 was found to be dependent on the signaling components of GPR55, that is, Gq protein, Ca^2+^, CaMKK, and PI3K. In Apo^−/^^−^ mice, administration of CID16020046 ameliorated high-fat diet-induced atherosclerosis development. These results suggest that high-fat diet-induced GPR55 activation leads to the adhesion of monocytes to endothelial cells via induction of Mac-1, and CID16020046 blockage of GPR55 could suppress monocyte adhesion to vascular endothelial cells through suppression of Mac-1 expression, leading to protection against the development of atherosclerosis.

## 1. Introduction

Atherosclerosis is a major contributor to cardiovascular diseases, including heart failure, myocardial infarction, stroke, and claudication [1,2]. Monocyte adhesion to endothelium is an early event in atherosclerosis in blood vessels [3,4]. Subsequently, adherent monocytes penetrate into the intima and differentiate into macrophages, which become foam cells by accumulating modified forms of low-density lipoproteins (LDL) [5]. Chronic inflammation caused by inflammatory stimuli, such as oxidized LDL (oxLDL), accelerates monocyte adhesion to endothelial cells [3,4,5]. During inflammatory reaction, specific adhesion molecules on the surface of endothelial cells (VCAM-1, ICAM-1, P-selectin, and E-selectin) and monocytes (Mac-1, LFA1, VLA-4, and PSGL-1) mediate the initial loose contact and subsequent tight adhesion [6,7,8].

GPR55 is recognized as a receptor for lysophosphatidylinositols [9,10,11,12,13] and is also known as an atypical cannabinoid receptor, because a range of endogenous, plant-derived, and synthetic cannabinoid ligands, such as 2-arachidonoylglycerol, palmitoylethanolamide, Δ^9^-THC, O-1602, and AM251, can activate GPR55 [11,14,15]. GPR55 is highly expressed in human monocytes, which are important inflammatory cells during the initiation and progression of atherosclerosis [16]. Although pro-atherogenic functions of GPR55 in endothelial cells and macrophages have been studied [17,18], the role of GPR55 in monocyte adhesion and atherosclerosis development has not yet been investigated. Thus, in this study, the stimulatory roles of GPR55 in atherosclerosis, especially monocyte adhesion to the vascular endothelium and high-fat diet (HFD)-induced plaque formation, were investigated. For this purpose, we employed monocyte adhesion to human umbilical vein endothelial cells (HUVECs) and HFD-induced atherosclerosis development in ApoE knockout (KO) mice along with O-1602 as a GPR55 agonist [15,19] and CID16020046 as a specific GPR55 antagonist [20].

## 2. Results

### 2.1. O-1602 Induced Adhesion of THP-1 Monocytes to HUVECs

**Figure 1:**
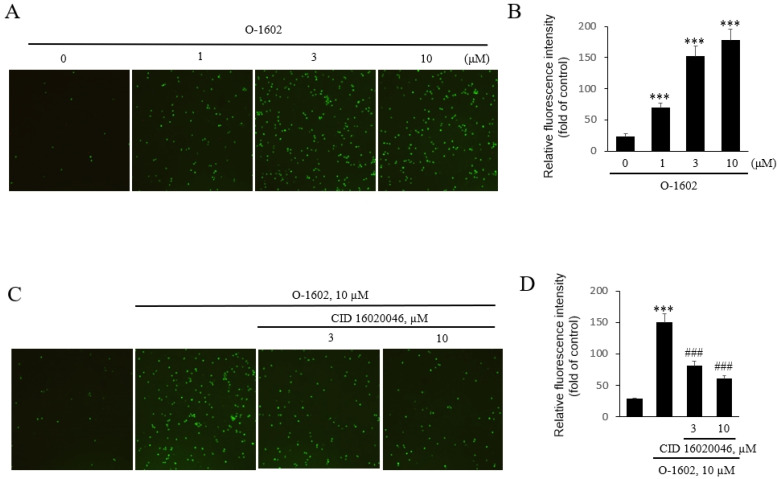
Effects of a GPR55 agonist, O-1602, on THP-1 monocytes adhesion to endothelial cells. THP-1 cells were treated with the indicated concentration of O-1602 for 24 h and labeled with 0.2 mg/L calcein-AM for 30 min at 37 °C. The labeled cells were seeded onto confluent HUVECs and images were obtained after 2 h using an inverse optical microscope (Axiovert 25) and Axio Vision Release 4.7 software (**A**) Representative fluorescence microscopic images of THP-1 monocyte adhesion to endothelial cells. (**B**) Histogram of THP-1 monocyte adhesion to endothelial cells THP-1 cells were treated with the indicated concentration of O-1602 for 24 h. (**C**,**D**) THP-1 cells were treated with CID16020046 for 1 h and then with the indicated concentration of O-1602 for 24 h. (**C**) Representative fluorescence microscopic images of THP-1 monocyte adhesion to endothelial cells. (**D**) Histogram of THP-1 monocyte adhesion to endothelial cells. The results are representative of four independent experiments. The results are presented as the mean ± SEM of four independent experiments. Statistical significance: *** *p* < 0.01 vs. untreated control cells, ### *p* < 0.001 vs. O-1602-treated cells.

Monocyte adhesion to endothelial cells is a key stage in the initiation of atherosclerosis, and adhesion is dependent on the interaction between adhesion molecules on monocytes and endothelial cells [4,5]. To investigate the role of GPR55 in monocyte adhesion to endothelial cells, THP-1 human monocytic cells were treated with O-1602 (a selective GPR55 agonist) and co-cultured in HUVECs. Treatment with O-1602 induced the adhesion of THP-1 monocytes to HUVECs in a dose-dependent manner (Figure 1A,B). Monocyte adhesion to endothelial cells induced by O-1602 was inhibited by CID16020046 (a selective GPR55 antagonist) in a concentration-dependent manner (Figure 1C,D), suggesting that GPR55 is involved in monocyte adhesion to endothelial cells.

### 2.2. Involvement of Gq/11 Proteins, Ca^2+^, CaMKK, and PI3K in GPR55-Mediated Monocyte Adhesion

**Figure 2:**
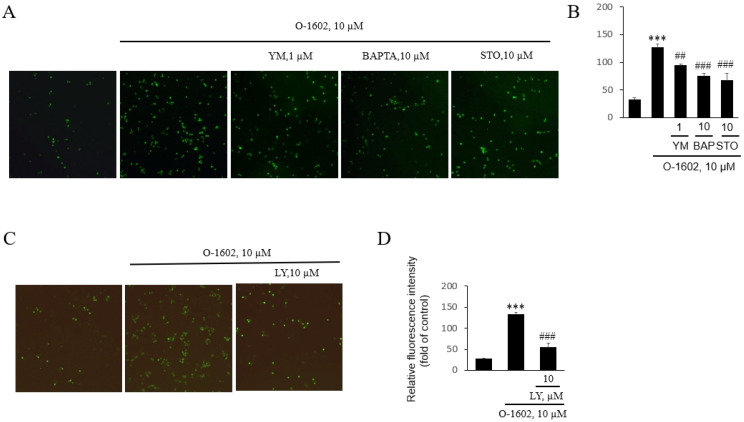
Effects of YM254890, BAPTA-AM, STO-609, and LY294002 on O-1602-induced THP-1 monocytes adhesion to endothelial cells. THP-1 cells were pretreated with the indicated concentrations of inhibitors for 1 h, and then treated with 10 μM O-1602 for 24 h. The calcein-labeled cells were seeded onto confluent HUVECs and images were obtained after 2 h. (**A**) Representative fluorescence microscopic images of THP-1 monocyte adhesion to HUVECs in the presence of YM254890, BAPTA-AM, and STO-609. (**B**) Histogram of THP-1 monocyte adhesion to endothelial cells in the presence of YM254890, BAPTA-AM, and STO-609. (**C**) Representative fluorescence microscopic images of THP-1 monocyte adhesion to HUVECs treated with LY294002. (**D**) Histogram of the adhesion of THP-1 monocytes to endothelial cells with LY294002. The results are presented as the mean ± SEM of three independent experiments. Statistical significance: *** *p* < 0.001 vs. untreated control cells, ## *p* < 0.01, ### *p* < 0.001 vs. O-1602-treated cells.

We explored how GPR55 activation leads to monocyte adhesion to HUVECs. The signaling components of GPR55 activation have been reported to be Gq proteins, phospholipase C, an increase in cytosolic Ca^2+^ concentration, and subsequent protein kinases [10,11,21]. Therefore, we utilized YM254890 (an inhibitor of Gq proteins), BAPTA-AM (an intracellular Ca^2+^ chelator), STO609 (an inhibitor of the Ca^2+^/calmodulin-dependent protein kinase kinase (CaMKK)), and LY294002 (an inhibitor of PI3K). As shown in Figure 2A,B, the signaling pathway of Gq/Ca^2+^/CaMKK was found to be partly involved in GPR55-mediated monocyte adhesion. That is, O-1602 induced monocyte adhesion was suppressed by the presence of YM254890, BAPTA, and STO-609 (Figure 2A,B). Additionally, the increased adhesion of THP-1 cells by O-1602 treatment was inhibited by LY294002 (Figure 2C,D). Therefore, it is suggested that GPR55 activation leads to the signaling cascade of Gq/Ca^2+^/CaMKK and PI3K for the induction of monocyte adhesion to endothelial cells.

### 2.3. Activation of GPR55-Induced Mac-1 Expression in THP-1 Cells

**Figure 3:**
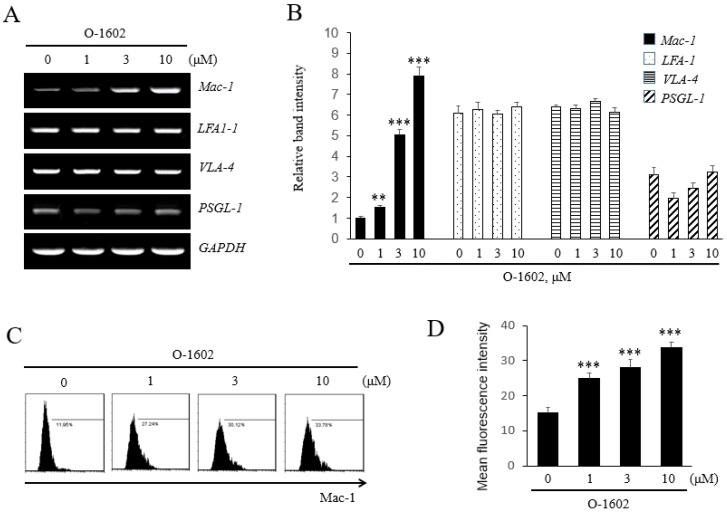
Effects of O-1602 on the expression of adhesion molecules in THP-1 cells. THP-1 cells were treated with the indicated concentration of O-1602 for 24 h. The mRNA expression of adhesion molecules was analyzed by RT-PCR. (**A**) Representative RT-PCR images. (**B**) Histograms showing the relative mRNA levels. (**C**) Representative flow cytometry images of Mac-1 protein. (**D**) Histograms showing relative Mac-1 protein levels. The results are presented as the mean ± SEM of three independent experiments. Statistical significance: ** *p* < 0.01, *** *p* < 0.001 vs. untreated control cells.

**Figure 4:**
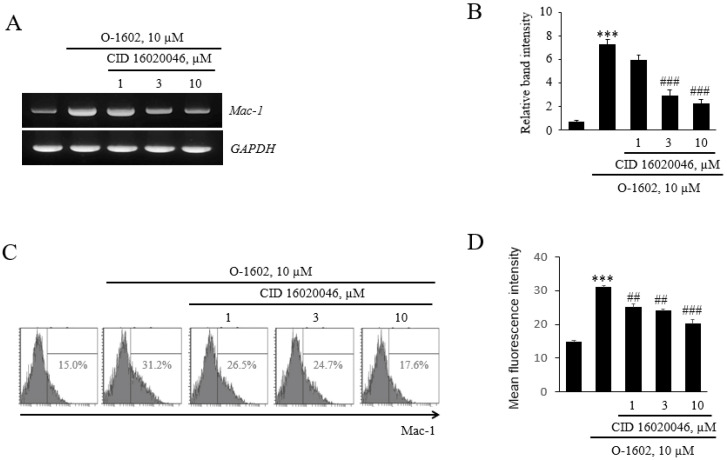
Effects of CID16020046 on GPR55-mediated Mac-1 expression in THP-1 cells. THP-1 cells were pretreated with the indicated concentrations of CID16020046 for 1 h, and then treated with 10 μM O-1602 for 24 h. The mRNA expression of adhesion molecules was analyzed by RT-PCR and flow cytometry. (**A**) Representative RT-PCR images. (**B**) Histograms showing the relative mRNA levels. (**C**) Representative flow cytometry images of Mac-1 proteins. (**D**) Histograms showing relative Mac-1 protein levels. The results are presented as the mean ± SEM of three independent experiments. Statistical significance: *** *p* < 0.001 vs. untreated control cells, ## *p* < 0.01, ### *p* < 0.001 vs. O-1602-treated cells.

Next, we investigated how GPR55 activation induces monocyte adhesion by examining the expression of adhesion molecules in THP-1 monocytes. Among adhesion molecules of the monocyte membrane, GPR55 activation induced *Mac-1*, but not other adhesion molecules, such as *LFA1-1*, *VLA-4*, and *PSGL-1* (Figure 3A), and the induction of *Mac-1* was found to be concentration-dependent (Figure 3B). In order to confirm the effect of GPR55 activation on the mRNA expression of *Mac-1*, its protein levels were checked by flow cytometry. It was found that O-1602 induced the protein expression of Mac-1 (Figure 3C), and the induction of Mac-1 was concentration-dependent (Figure 3D). Pretreatment with CID16020046 suppressed the O-1602-induced mRNA expression and protein expression of Mac-1 in a concentration-dependent manner (Figure 4), demonstrating the involvement of GPR55 in Mac-1 expression in monocytes.

### 2.4. Involvement of Gq/11 Proteins, Ca2+, CaMKK, and PI3K in GPR55-Mediated Mac-1 Expression

**Figure 5:**
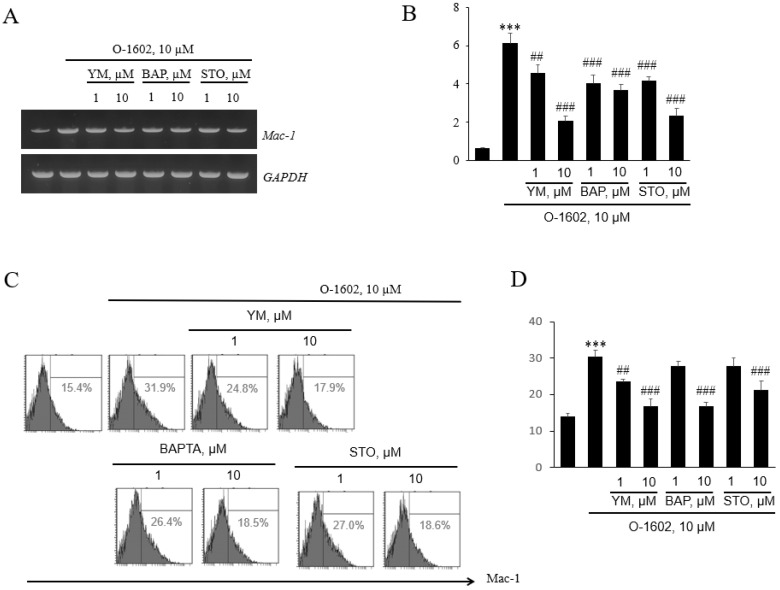
Effects of YM254890, BAPTA-AM, and STO-609 on O-1602-induced Mac-1 expression in THP-1 cells. THP-1 cells were pretreated with the indicated concentrations of inhibitors for 1 h, and then treated with 10 μM O-1602 for 24 h. The mRNA expression of adhesion molecules was analyzed by RT-PCR and flow cytometry. (**A**) Representative RT-PCR images. (**B**) Histograms showing the relative mRNA levels. (**C**) Representative flow cytometry images of Mac-1 proteins. (**D**) Histograms showing relative Mac-1 protein levels. The results are presented as the mean ± SEM of three independent experiments. Statistical significance: *** *p* < 0.001 vs. untreated control cells, ## *p* < 0.01, ### *p* < 0.001 vs. O-1602-treated cells.

We investigated whether the signaling components of GPR55 lead to Mac-1 expression in monocytes using pharmacological inhibitors. As shown in Figure 5A,B, GPR55-mediated expression of *Mac-1* mRNA was suppressed by the presence of YM254890, BAPTA, and STO-609 (Figure 5A,B). These inhibitions were further confirmed at the protein level by flow cytometry (Figure 5C,D).

**Figure 6:**
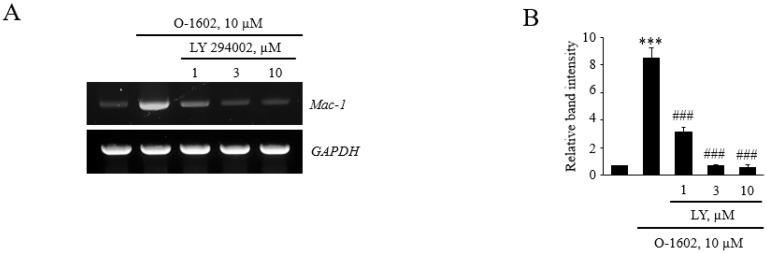

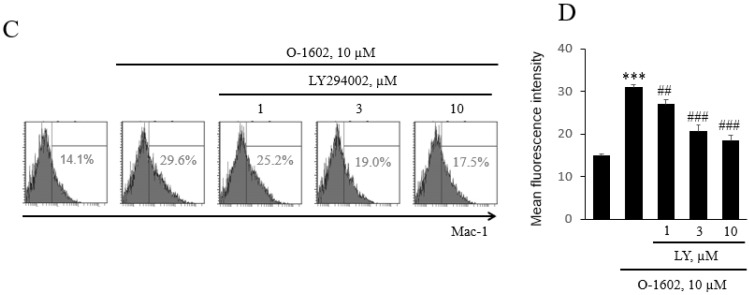
Effects of LY294002 on O-1602-induced Mac-1 expression in THP-1 cells. THP-1 cells were pretreated with the indicated concentrations of LY294002 for 1 h, and then treated with 10 μM O-1602 for 24 h. mRNA expression of adhesion molecules was analyzed by RT-PCR and flow cytometry. (**A**) Representative RT-PCR images. (**B**) Histograms showing the relative mRNA levels. (**C**) Representative flow cytometry images of Mac-1 proteins. (**D**) Histograms showing relative Mac-1 protein levels. The results are presented as the mean ± SEM of three independent experiments. Statistical significance: *** *p* < 0.001 vs. untreated control cells, ## *p* < 0.01, ### *p* < 0.001 vs. O-1602-treated cells.

Additionally, Mac-1 expression by O-1602 treatment in monocytes was inhibited by LY294002 at the mRNA and protein levels (Figure 6). Therefore, GPR55 activation induces Mac-1 expression through signaling cascades of Gq/Ca^2+^/CaMKK and PI3K in monocytes.

### 2.5. Cloning and Characterization of the Mac-1 Promoter Involved in O-1602-Induced Mac-1 Transcription

**Figure 7:**
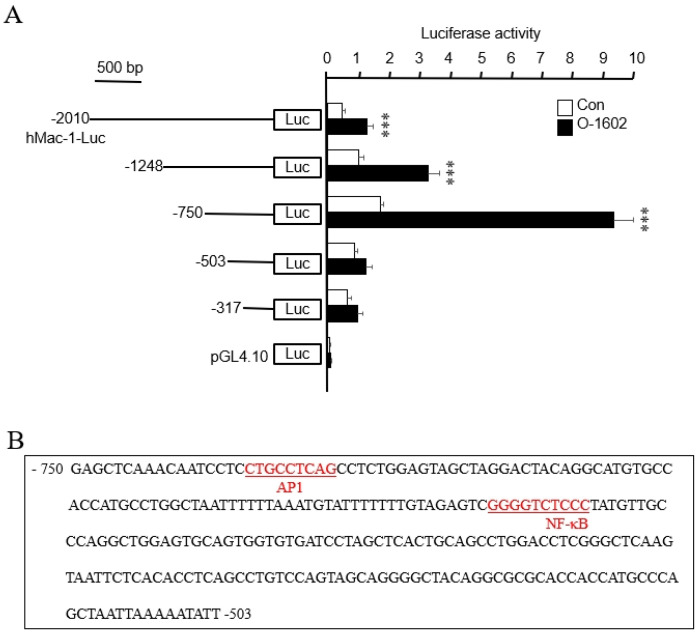
Cloning and characterization of the *Mac-1* promoter involved in GPR55-mediated *Mac-1* transcription. (**A**) THP-1 cells were co-transfected transiently with various promoter constructs and an empty luciferase vector pGL4.10 for 24 h, and then treated with or without 10 μM O-1602 for 24 h. The relative luciferase activity was presented as means ± SE of five independent experiments. Statistical significance: *** p < 0.001 vs. untreated control cells. (**B**) Nucleotide sequence of the promoter region of the Mac-1 gene. The sequence of the region between nt −750 and −503 bp of the 5′-flanking region is shown. The underlined sequences are possible transcription factor binding sites, as predicted by TFSEARCH.

To locate the region responsible for GPR55-induced *Mac-1* transcription, luciferase activity was conducted after transiently transfecting six constructs with different sizes of the 2010 nt promoter region of *Mac-1*. As shown in Figure 7A, the luciferase reporter activity of pMac1-2010 was higher in several constructs after O-1602 treatment than that in the control. Among five constructs, the promoter containing nucleotides from nt −750 to nt −503 showed the strongest induction in response to O-1602 (Figure 7A). This suggests that the region between nt −750 and nt −503 is responsible for GPR55-induced *Mac-1* transcription in monocytes. Sequence analysis within this region demonstrated the presence of consensus elements for transcription factors, including AP-1 and NF-κB (Figure 7B).

### 2.6. Involvement of AP-1 and NF-κB in GPR55-Induced Mac-1 Transcription

**Figure 8:**
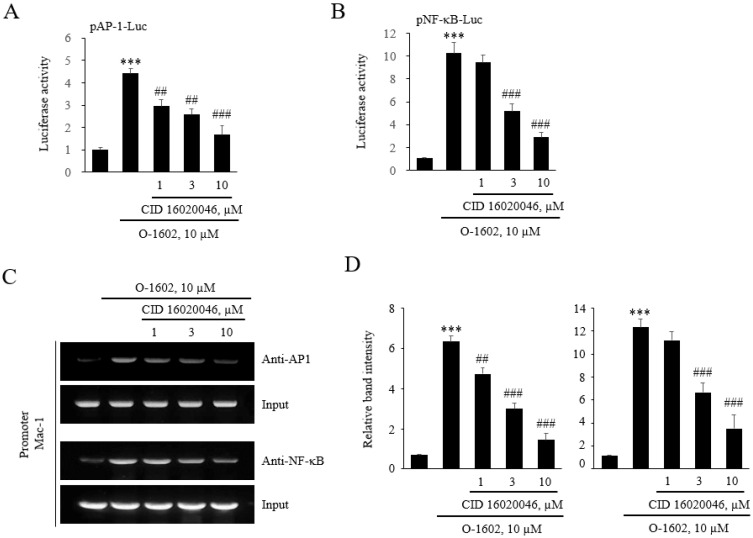
Involvement of AP-1 and NF-κB in GPR55-mediated *Mac-1* transcription. (**A**,**B**) THP-1 cells were transfected with luciferase reporter constructs containing AP-1 or NF-κB consensus binding sites, pretreated with the indicated concentrations of CID16020046 for 1 h, and then treated with 10 μM O-1602 for 24 h. The AP-1 (**A**) and NF-κB (**B**) luciferase activities were represented as the mean ± SEM from five to six independent experiments. (**C**,**D**) Binding of AP-1 and NF-κB to the *Mac-1* promoter was detected with the chromatin immunoprecipitation (ChIP) assay. Immunocomplexes of AP-1 and NF-κB associated with DNA were obtained from THP-1 cells. The THP-1 cells were pretreated with the indicated concentrations of CID16020046 for 1 h, and then treated with 10 μM O-1602 for 24 h. Specific DNA fragments were quantified by PCR, as detailed in the Methods section. DNA purified from lysates incubated without antibody was used as input control (Input). Representative images (**C**) and quantitated histograms of the ChIP assay. The results are presented as means ± SEM of three independent experiments. Statistical significance: *** *p* < 0.001 vs. non-treated control cells, ## *p* < 0.01 and ### *p* < 0.001 vs. O-1602-treated cells.

To assess whether GPR55 activation induced binding of AP-1 and NF-κB to the responsive sites, luciferase constructs containing tandem repeats of consensus sequences of AP-1 or NF-κB were used. The O-1602 treatment induced an increase in luciferase activity in pAP-1-Luc and pNF-κB-Luc, and these increases were suppressed by the CID16020046 treatment (Figure 8A,B), confirming that GPR55 activation leads to Mac-1 transcription via binding of AP-1 and NF-κB to the responsive sites in the promoter region of *Mac-1*. Next, we performed ChIP assays to directly assess whether GPR55 activation induced binding of AP-1 and NF-κB to the binding sites of the *Mac-1* promoter region. After nuclear extraction from monocytes treated with O-1602 or O-1602 plus CID16020046, protein-DNA complexes were immunoprecipitated with anti-AP-1 or anti-NF-κB antibodies. Subsequent PCR amplification of the *Mac-1* promoter showed increases in AP-1 and NF-κB binding to the Mac-1 promoter after the O-1602 treatment (Figure 8C). The increased binding was suppressed by CID16020046 treatment in a dose-dependent manner (Figure 8C,D), demonstrating that the binding of AP-1 and NF-κB participates in GPR55-mediated induction of *Mac-1* expression.

### 2.7. Involvement of Gq/11 Proteins, Ca^2+^, CaMKK, and PI3K in GPR55-Induced Inductions of AP-1 and NF-κB

**Figure 9:**
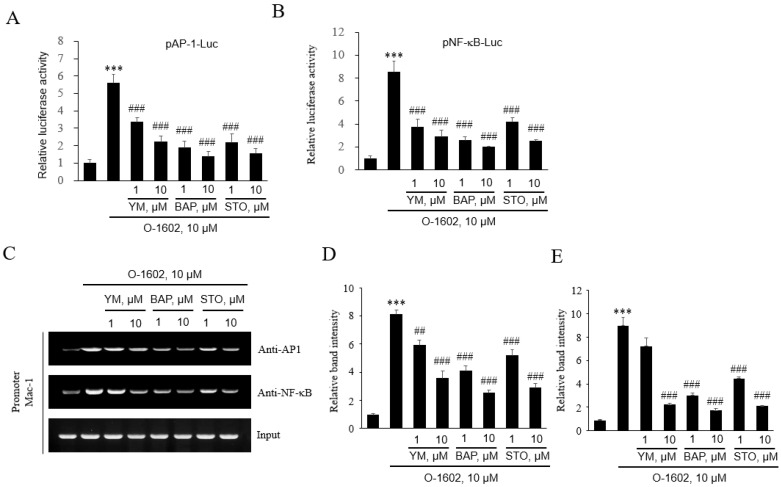
Effects of YM254890, BAPTA-AM, and STO-609 on O-1602-induced expression of AP-1 and NF-κB in THP-1 cells. THP-1 cells were pretreated with the indicated concentrations of inhibitors for 1 h, and then treated with 10 μM O-1602 for 24 h. Mac-1 mRNA, promoter activity, and protein expression were analyzed by reporter gene assay and chromatin immunoprecipitation (ChIP) assay. The AP-1 (**A**) and NF-B (**B**) luciferase activities are represented as the mean ± SEM from five to six independent experiments. (**C**–**E**) Binding of AP-1 and NF-κB to the Mac-1 promoter was detected by ChIP assay. Representative images (**C**) and quantitative histograms of the ChIP assay (D for AP-1 and E for NF-κB). The results are presented as the mean ± SEM of three independent experiments. Statistical significance: *** *p* < 0.001 vs. untreated control cells, ## *p* < 0.01 and ### *p* < 0.001 vs. O-1602-treated cells.

**Figure 10:**
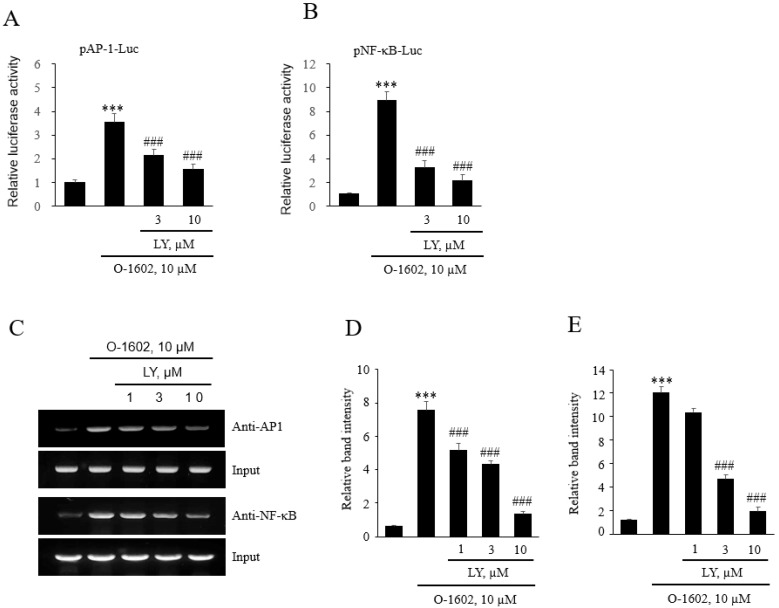
Effects of LY294002 on O-1602-induced expression of *AP-1* and *NF-κB* in THP-1 cells. THP-1 cells were pretreated with the indicated concentrations of LY294002 for 1 h, and then treated with 10 μM O-1602 for 24 h. *Mac-1* mRNA, promoter activity, and protein expression were analyzed by reporter gene assay and chromatin immunoprecipitation (ChIP) assay. The AP-1 (**A**) and NF-B (**B**) luciferase activities are represented as the mean ± SEM from five to six independent experiments. (**C**–**E**) Binding of AP-1 and NF-κB to the *Mac-1* promoter was detected by ChIP assay. Representative images (**C**) and quantitative histograms of the ChIP assay (D for AP-1 and E for NF-κB). The results are presented as the mean ± SEM of three independent experiments. Statistical significance: *** *p* < 0.001 vs. untreated control cells, ### *p* < 0.001 vs. O-1602-treated cells.

We investigated the signaling components of GPR55-induced induction and binding of AP-1 and NF-κB to the promoter region of Mac-1. The same signaling components of GPR55 activation, that is, Gq proteins, phospholipase C, increase in cytosolic Ca^2+^ concentration, and subsequent protein kinases, were studied with inhibitors of YM254890, BAPTA-AM, STO609, and LY294002. As shown in Figure 9, the signaling pathway of Gq/Ca^2+^/CaMKK was found to be involved in the GPR55-mediated induction of AP-1 and NF-κB, which was demonstrated by the fact that O-1602-induced increase of luciferase activities in pAP-1-Luc and pNF-κB-Luc was suppressed by the presence of inhibitors (YM254890, BAPTA-AM, and STO609) (Figure 9A,B). Additionally, increased binding of AP-1 and NF-κB to the *Mac-1* promoter region was found to be mediated through the signaling pathway of Gq/Ca^2+^/CaMKK in the ChIP assays (Figure 9C–E). Similarly, the presence of LY294002 suppressed GPR55-mediated induction of AP-1 and NF-κB (Figure 10A,B). Furthermore, direct binding of AP-1 and NF-κB to the Mac-1 promoter region by GPR55 activation was also suppressed by LY294002 in the ChIP assays (Figure 9C–E). Therefore, GPR55 activation is suggested to signal the cascade of Gq/Ca^2+^/CaMKK and PI-3K for the induction of AP-1 and NF-κB and direct binding of AP-1 and NF-κB to the *Mac-1* promoter region in monocytes.

### 2.8. Inhibition of GPR55 Ameliorated Atherosclerosis Development in an ApoE KO Mouse Model

**Figure 11:**
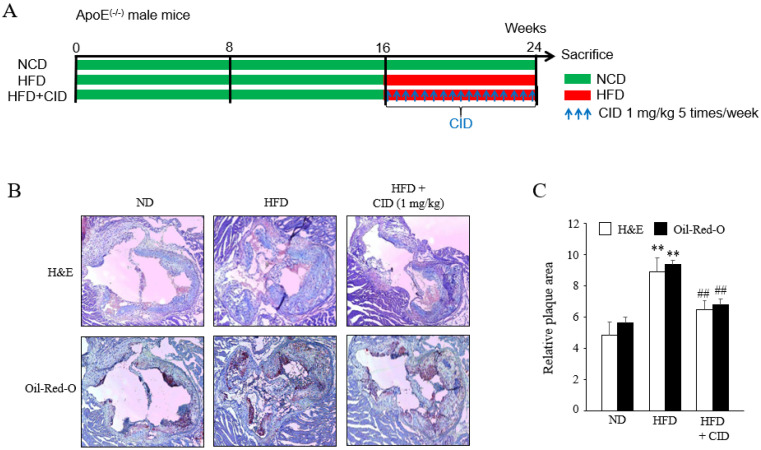
Effects of GPR55 blockage on atherosclerotic plaque formation in vivo. (**A**) High fat-diet (HFD)-induced atherosclerosis protocol. Sixteen-week aged *ApoE*^−/−^ mice were followed up for 8 weeks under normal chow diet (ND) or were fed with an HFD from 16 weeks to 24 weeks (HFD). Another group of mice, that is, the HFD plus CID16020046 group, was intraperitoneally treated with 1 mg/kg CID16020046, five times per week during HFD feeding before euthanasia. (**B**) Images of H&E and oil red O staining in cross-sections of the aortic sinuses from the ND, HFD, or HFD plus CID16020046 groups. Representative images from five or six independent mice are shown. (**C**) Quantitative data are presented as the means ± SEM of five or six independent mice. ** *p* < 0.01 compared with an ND, ## *p* < 0.01 compared with an HFD.

Based on the results of the in vitro cell experiments, a high-fat diet (HFD)-induced model of atherosclerosis development was used in ApoE KO mice. Male ApoE KO mice were fed a normal diet (ND) for 16 weeks. Mice were then fed an ND or HFD for 8 weeks (Figure 11A). Another group of mice, that is, the HFD plus CID16020046 group, was fed the HFD for 8 weeks, and at the same time, treated with CID16020046 (1 mg/kg, i.p., five times per week) for 8 weeks (Figure 11A). The HFD induced an increase in the body weight and plasma glucose level by 30% and 64%, respectively, but decreased HDL cholesterol levels by 41% (Table 1). The HFD induced an increase in the plasma total cholesterol, LDL cholesterol, and triglycerides by 290%, 401%, and 159%, respectively (Table 1). CID16020046 administration suppressed the increase in body weight and plasma glucose level by 86% and 66%, respectively, but increased HDL levels by 127%. Furthermore, CID16020046 administration suppressed the increase in plasma total cholesterol, LDL cholesterol, and triglycerides by 79%, 81%, and 84%, respectively. Therefore, inhibition of GPR55 improved all the harmful parameters induced by HDF, that is, body weight, glucose level, total cholesterol, LDL cholesterol, HDL cholesterol, and triglycerides. These improvements were further confirmed by histological analysis of the atherosclerotic plaques. As shown in Figure 11B,C, narrowed vessels and increased plaque thickness were observed in the HFD group, and CID16020046 treatment inhibited vessel narrowing and plaque thickening. Oil red O staining showed severe lipid accumulation in the plaque of HFD-fed mice and reduction of lipid accumulation in the plaque of CID16020046-treated mice (Figure 11B).

## 3. Discussion

**Figure 12:**
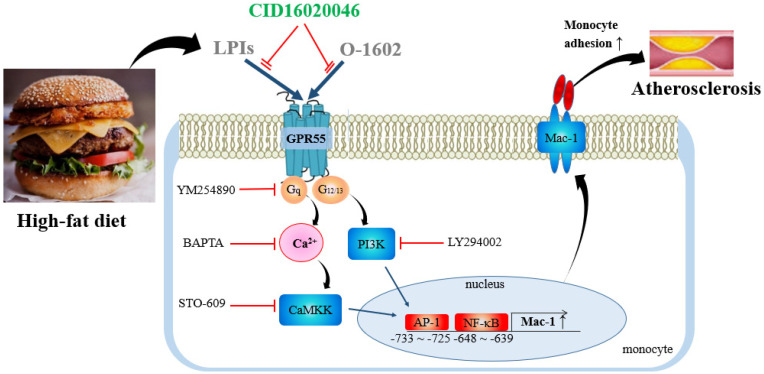
Proposed mechanism of GPR55 activation in monocyte adhesion and atherosclerosis development. Activation of GPR55 leads to Mac-1 induction through AP-1 and NF-κB by signaling cascades of Gq/Ca^2+^/CAMKK and PI3K. Increased Mac-1 expression enhances monocyte adhesion and atherosclerosis development. LPI, lysophopshatidylinositol.

In this study, we reported the following: (1) GPR55 activation by O-1602 induced monocyte adhesion to endothelial cells via Mac-1 expression; (2) Mac-1 expression is regulated by binding of the transcription factors AP-1 and NF-κB to the nt −750 to −503 promoter region of *Mac-1* in monocytes, (3) signaling cascades of GPR55 activation to *Mac-1* gene expression are Gq/Ca^2+^/CaMKK and PI-3K, and (4) blockage of GPR55 protected against atherosclerosis progression along with improved lipid profiles, as shown in Figure 12. Therefore, our results are associated with several points, including monocyte adhesion, atherosclerosis protection, and improved lipid profiles.

The adhesion of monocytes to endothelial cells by GPR55 activation has been extensively studied. This not only confirms the signaling cascade of GPR55 but also elucidates the adhesion molecule of *Mac-1* expression and its promoter analysis. During the study, we found that O-1602-induced cell adhesion appears to result from the upregulation of *Mac-1* surface expression in monocyte cells, making a firm association with ICAM-1 expression on the vascular endothelium [18]. Our results clearly showed that O-1602 induced *Mac-1* mRNA and protein expression, as well as the activity of the *Mac-1* promoter. Based on the experimental results and other reports on Mac-1 expression at the transcriptional level [22,23], we tried to identify the essential regulatory elements in the promoter region of the *Mac-1* gene. We found that the essential region between nt −750 and −503 bp contains consensus binding sites for AP-1 and NF-κB, and the ChIP assays confirmed that O-1602 enhanced binding of AP-1 and NF-κB to the corresponding binding sites of the *Mac-1* promoter. Thus, we discovered that AP-1 and NF-κB are essential transcription factors for O-1602-induced induction of *Mac-1* transcription and subsequent monocyte adhesion.

The blockage of GPR55 by CID16020046 in vivo protects against atherosclerosis progression. The effect of CID16020046 on monocyte adhesion may partly explain the protection against atherosclerosis progression. Furthermore, previous studies have supported the in vivo results. For example, mRNA and protein levels of GPR55 were detected in human macrophages, and their expression were increased in foam cells [17]. Activation of GPR55 by O-1602 exacerbated oxLDL-induced lipid accumulation by upregulating CD36 and SR-BI, while it reduced cholesterol efflux by downregulating ABC-A1 and ABC-G1 in human macrophages [17]. Activation of GPR55 by O-1602 also induced pro-inflammatory TNF-α and reduced anti-inflammatory IL-10 expression in human macrophages [17]. Therefore, blockage of GPR55 in macrophages and foam cells may suppress lipid accumulation and inflammatory responses in atherosclerotic plaques, strongly supporting the in vivo results of the present study. Furthermore, oxLDL increased GPR55 expression in human aortic endothelial cells [18]. CID16020046 protects against oxLDL-induced cell death, ROS generation, and secretion of pro-inflammatory cytokines such as IL-8 and MCP-1 in human aortic endothelial cells [18]. CID16020046 also suppressed oxLDL-induced expression of adhesion molecules VCAM-1 and E-selectin in human aortic endothelial cells [18]. These results from endothelial cells also support the pro-atherosclerotic roles of GPR55, which is consistent with the present findings of GPR55 blockage-induced protection against atherosclerosis progression.

Montecucco et al. reported that the CID16020046 treatment did not affect atherogenesis [24]. However, several points of CID16020046 treatment differed between the report and our experiment. They used a 0.5 mg/kg dose for 3 weeks in an 11-week high-cholesterol diet protocol, while we used a 1 mg/kg dose five times per week in an 8-week HFD protocol. We used a two-fold higher dose than the study of Montecucco et al. (1 mg/kg vs. 0.5 mg/kg) and used an HFD instead of high-cholesterol diets [24]. Therefore, a dose of 1 mg/kg seems to be optimal to achieve efficacy in vivo, which has recently been supported in an HFD-induced fatty liver model [25]. In addition, high-cholesterol diets were inadequate to activate GPR55, because lysophosphoinositols, the endogenous ligands of GPR55, are provided in an HFD but not in high-cholesterol diets [25,26]. Especially, levels of lysophosphatidylinositols of 18:0, 18:1, 18:2, and 18:3 were significantly increased in the liver after HFD feeding [25]. Additionally, an HFD may provide other lipid agonists of GPR55, such as oleoylethanolamide and lysophosphatidylcholines [26,27,28,29].

Blockage of GPR55 by CID16020046 also improved lipid profiles in vivo. This may be due to the blockage of GPR55 in hepatocytes and adipocytes. Indeed, CID16020046 suppressed HFD-induced hepatic steatosis in mice, suggesting that increased lysophosphatidylinositols may be a cause of hepatic steatosis in overnutrition conditions [25]. In fact, serum levels of lysophosphatidylinositol species of 16:0, 18:1, and 18:1 isomers were found to be high in patients with non-alcoholic steatohepatitis [30]. Circulating lysophosphatidylinositol levels are increased in patients with non-alcoholic fatty liver disease [30]. Lysophosphatidylinositol augments lipid content by inducing de novo lipogenesis and decreasing β-oxidation [30]. In addition, lysophosphatidylinositol promotes the initiation of hepatic stellate cell activation by stimulating acetyl-coenzyme A carboxylase-α [30]. Similarly, the lysophosphatidylinositol-GPR55 system has been positively associated with human obesity [31]. Lysophosphatidylinositols increased the expression of lipogenic genes (*fatty acid synthase* and *acetyl CoA carboxylase*) and promoted adipocyte differentiation by increasing PPAR expression in visceral adipose tissues [28]. Furthermore, acute O-1602 administration induced hyperphagia, which was accompanied by decreased expression of the anorexigenic neuropeptide CART, and chronic O-1602 administration increased adiposity [32]. Increased expression of GPR55 in visceral adipose tissue and liver of obese patients might support the results of the present study and suggest GPR55 as a possible therapeutic target for improving lipid profiles [30,31].

In summary, this study provides important evidence that GPR55 blockage markedly inhibits HFD-induced atherosclerosis development and improves HFD-induced lipid profiles. Mechanistically, GPR55-mediated induction of *Mac-1* expression was found to be regulated at the transcriptional level in monocytes by activation of the AP-1 and NF-κB pathways through Gq/Ca^2+^/CaMKK and PI-3K. Collectively, these results suggest that GPR55 activation might be the key component responsible for the pro-atherosclerotic effects of the HFD.

## 4. Materials and Methods

### 4.1. Chemicals and Antibodies

O-1602 and CID16020046 were purchased from Tocris Bioscience (Ellisville, MO, USA). R-phycoerythrin (PE)-conjugated mouse anti-human Mac-1 (clone ICRF44; BD) antibody (Cat No. 555388) and PE-conjugated mouse IgG isotype control (clone MOPC-21) antibody (Cat No. 555749) were obtained from BD Biosciences (San Diego, CA, USA). Various signal pathway inhibitors were purchased from EMD Serono (Rockland, MA, USA) and Sigma-Aldrich (St. Louis, MO, USA).

### 4.2. Cell Culture

THP-1 (a human monocytic leukemia cell line) cells were purchased from ATCC (Manassas, VA, USA). Cells were grown in RPMI 1640 medium (Life Technologies, Carlsbad, CA, USA) supplemented with 10% heat-inactivated fetal bovine serum (FBS), antibiotic-antimycotic, and L-glutamine (Life Technologies). The HUVECs were obtained from Lonza Walkersville (Walkersville, MD, USA) and cultured in endothelial growth medium-2 (EGM-2 MV; Lonza). The cells were maintained at 37 °C in a humidified atmosphere containing 5% CO_2_ and 95% air.

### 4.3. Animals and Diets

The ApoE KO mice were purchased from Jackson Laboratories. Animals were allowed free access to tap water and laboratory rodent chow at 20–22 °C and 50–60% relative humidity. All animal procedures conformed to the Guide for the Care and Use of Laboratory Animals published by the US National Institute of Health (NIH Publication No. 85-23, revised 1996), and the experimental protocols were approved by the Pusan National University Institutional Animal Care and Use Committee. In this study, 16-week-old mice were randomly divided into three groups: control (n = 5), in which mice were fed a normal chow for 8 weeks; HFD, in which mice (n = 6) were fed an HFD for 8 weeks; and HDF plus CID16020046, in which mice (n = 5) were fed an HDF for 8 weeks with CID16020046 (1 mg/kg) five days per week for the same 8 weeks (experimental scheme in Figure 11). The animal protocol used in this study was reviewed and approved by the PNU Institutional Animal Care Committee (PNU–IACUC) for compliance with the ethics of the procedures and animal care.

### 4.4. Flow Cytometric Analysis

To determine Mac-1 protein expression, THP-1 cells (1 × 105/mL) were collected from cultures and washed with fluorescence-activated cell sorting (FACS) buffer (PBS containing 1% FCS and 0.05% NaN3). Cells were then incubated with FcR blocker (anti-human IgG; Sigma-Aldrich Co.) to block nonspecific antibody binding, and bound with PE-conjugated mouse anti-human Mac-1 (clone ICRF44; BD) with matched pairs of PE-conjugated mouse IgG isotype control (clone MOPC-21) antibody. Analysis was performed using FACSCalibur and CELLQUESTPRO software (BD), which recorded 10,000 cells in each individual sample. Live cells were gated based on size (FSC) and granularity (SSC), and Mac-1 expression was analyzed.

### 4.5. Adhesion Assay

The THP-1 monocytes were labeled with 0.2 mg/L calcein-AM for 30 min at 37 °C, and labeled cells were seeded onto confluent HUVECs. After 2 h, co-cultured cells were washed with 1× PBS containing 1% bovine serum albumin (BSA), and images were obtained using an inverse optical microscope (Axiovert 25) and Axio Vision Release 4.7 software (Carl Zeiss MicroImaging GmbH, Oberkochen, Germany). Localization data were quantified using ImageJ analysis.

### 4.6. RNA Isolation and RT-PCR

Total RNA was isolated using TRIzol reagent (Life Technologies) and reverse transcribed using the Improm II reverse transcription system. The reverse transcribed cDNA was amplified by PCR using specific primers and conditions as previously described [33]. PCR products were separated on 1.2% agarose gels and stained with ethidium bromide solution.

### 4.7. Preparation of Mac-1 Promoter Constructs

Human genomic DNA was isolated from human macrophages using a DNeasy Tissue Kit. The 2 kb 5′-flanking promoter region from the genomic DNA was amplified by PCR using the upstream primer, 5′-ATT GGC GGT ACC ATA AAG GTG AGG TTT GTG-3′, and the downstream primer, 5′-ATG CAC CTG CTA GCA GAA GGA CTC TCA GAG-3′; underlined are the sites of the restriction enzymes KpnI and NheI. Both primers were designed based on sequences retrieved from the GenBank Accession J03925. The amplified 2010 bp fragment was cloned into the luciferase vector pGL4.10 Basic (Promega, Fitchburg, WI, USA). Additional deletion constructs lacking distal promoter sequences (denoted pMac1–Luc-1248, pMac1–Luc-750, pMac1–Luc-503, and pMac1–Luc-317) were prepared by the digestion of pMac1–Luc-2010 with the restriction enzymes BglII, SacI, SspI, or NdeI. The Mac-1 promoter sequence was analyzed for any transcription factor binding sites within the 5′-flanking promoter region using the sequence motif search program devised by TFresearch (http://mbs.cbrc.jp/research/db/TFSEARCH.html (accessed on 1 March 2021)).

### 4.8. Chromatin Immunoprecipitation (ChIP) Assays

The ChIP analysis was performed using the Millipore ChIP kit (Millipore, Billerica, MA, USA), following the manufacturer’s instructions with minor modifications. For each assay, the THP-1 monocytes were inoculated into a 10-cm dish (a total of 5 × 106 cells) and fixed with 1% formaldehyde. Cell pellets were resuspended in SDS lysis buffer containing protease inhibitors (1 mM PMSF, 1 µg/mL aprotinin, and 1 µg/mL pepstatin A). The samples were sonicated with a Misonix sonicator 3000 (Misonix, Farmingdale, NY, USA), centrifuged, and diluted 10-fold in ChIP dilution buffer. After removing an aliquot (whole-cell extract input), the chromatin samples were incubated at 4 °C overnight with antibodies against AP-1 (#9165, Cell Signaling) or NF-κB (#8242, Cell Signaling). The samples were then precipitated by binding to protein A-agarose/salmon sperm DNA beads (Millipore, Billerica, MA, USA). The immunoprecipitated chromatin was analyzed by PCR using primers for the Mac-1 gene promoter. Cycling parameters were 58 °C for 1 min and 95 °C for 30 s, followed by 40 cycles.

### 4.9. Transient Transfection and Luciferase Assay

The THP-1 cells were grown to 90–95% confluence in 12-well plates. One microgram of plasmid DNA and 2 µL of Lipofectamine LTX reagent (Invitrogen, Carlsbad, CA, USA) were separately diluted in 50 µL of Opti-MEM medium (GIBCO, New York, NY, USA), mixed together, and incubated at room temperature for 30 min. Cells were then washed with serum-free medium before adding 400 µL of Opti-MEM medium, and then the diluted mixed solution was added to the cells. Plates were incubated at 37 °C for 6 h. Subsequently, the conditioned medium was removed, and the cells were grown in fresh medium containing 10% FBS for 24 h. Cells were untreated or treated with O-1602. Cell lysates were prepared using passive lysis buffer from Promega assay system (Promega, Madison, WI, USA) and were used to measure luciferase activity according to the manufacturer’s instructions for the dual luciferase reporter assay (Promega, Madison, WI, USA). All firefly luciferase values were normalized to Renilla luciferase to compare transfection efficiencies. The results are presented as the mean ± SEM of a representative experiment performed in triplicate.

### 4.10. Statistical Analysis

The results are expressed as the mean ± SEM of the number of experiments indicated in the figure legends. Statistical significance of differences was calculated using a one-way ANOVA with a Bonferroni post hoc test for multiple group comparisons or Student’s unpaired t-test for two-group comparisons, where appropriate. The analyses were performed using GraphPad Prism Software version 5.02 (GraphPad Inc., La Jolla, CA, USA). Statistical significance was set at *p* < 0.05.

## Figures and Tables

**Table 1:** Changes in body weight and plasma lipid profiles in mice.

	ND (n = 5)	NFD (n = 6)	HFD + CID (1 mg/kg) (n = 5)
Body weight, g	35.9 ± 0.8	46.7 ± 1.8 **	37.4 ± 1.4 ^##^
Glucose, mg/dL	174.8 ± 10.7	287.3 ± 22.8 **	212.7 ± 55.1 ^##^
Total cholesterol	319.3 ± 4.6	927.6 ± 45.9 ***	445.9 ± 41.7 ^###^
LDL cholesterol	212.6 ± 9.9	852.3 ± 57.7 ***	333.6 ± 55.6 ^###^
HDL cholesterol	88.9 ± 9.1	52.8 ± 9.7 **	98.7 ± 9.4 ^###^
Triglycerides	79.3 ± 12.0	126.0 ± 33.6 **	86.7 ± 19.6 ^##^

Statistical significance: ** *p* < 0.01, *** *p* < 0.001 vs. ND, ## *p* < 0.01, ### *p* < 0.001 vs. HFD.

## Abbreviations

LDL: low-density lipoproteins; VCAM-1: vascular cell adhesion molecule 1; ICAM-1: intercellular adhesion molecule 1; Mac-1: macrophage-1 antigen; LFA-1: lymphocyte function-associated antigen-1; VLA-4: very late antigen-4; PSGL-1: P-selectin glycoprotein ligand-1; THC: tetrahydrocannabinol; HFD: high-fat diet; HUVEC: human umbilical vein endothelial cells; KO: knockout; CaMKK: Ca^2+^/calmodulin-dependent kinase kinase; YM: YM254890; BAP: BAPTA-AM; STO: STO-609; LY: LY294002; NF-κB: nuclear factor k-light-chain-enhancer of activated B cells; MCP-1: monocyte chemoattractant protein 1.
